# Hotairm1 Controls S100A9 Protein Phosphorylation in Myeloid-Derived Suppressor Cells during Sepsis

**Published:** 2023-08-03

**Authors:** Isatou Bah, Dima Youssef, Zhi Q. Yao, Charles E. McCall, Mohamed El Gazzar

**Affiliations:** 1 Department of Internal Medicine, Infectious Disease and Immunity, East Tennessee State University College of Medicine, Johnson City, TN 37614, USA;; 2 Center of Excellence in Inflammation, Infectious Disease and Immunity, East Tennessee State University College of Medicine, Johnson City, TN 37614, USA;; 3 Department of Internal Medicine, Section of Molecular Medicine, Wake Forest University School of Medicine, Winston-Salem, NC 27157, USA

**Keywords:** Sepsis, Hotairm1, S100A9, MDSC, Immune suppression

## Abstract

During the acute phase of sepsis, the S100A9 proinflammatory protein resides in the cytosol in a phosphorylated form. In contrast, S100A9 relocalizes to the nucleus in an unphosphorylated form during the late/chronic sepsis state of immunometabolic paralysis. We reported that Hotairm1, a long noncoding RNA, facilitates S100A9 nuclear location in myeloid-derived suppressor cells. Here, we show that Hotairm1 promotes S100A9 nuclear location by limiting its phosphorylation by p38 MAPK. Knockdown of Hotairm1 in MDSCs from mice and humans with late sepsis increases phospho-S100A9 protein. Conversely, increasing Hotairm1 in early sepsis Gr1^+^CD11b^+^ cells by transfection decreases phospho-S100A9 protein levels. Notably, increasing S100A9 protein phosphorylation in late sepsis MDSCs *via* Hotairm1 knockdown decreases the production of the immunosuppressive cytokine IL-10. These results suggest that targeting Hotairm1 might reduce MDSC expansion during sepsis and thus relieve immunosuppression and improve survival.

## INTRODUCTION

The immune response to sepsis involves concurrent hyperinflammation and immune suppression [1–3]. While the hyperinflammatory response prevails during the early/acute phase of sepsis, many patients who survive this acute phase response develop a chronic critical illness phenotype characterized by persistent inflammation and immune suppression [4,5], which increases mortality and morbidity rates among patients suffering late/protracted sepsis.

Following sepsis initiation, inflammatory mediators IL-6, IL-1 and ROS induce phagocytes (mainly neutrophils) to mobilize from bone marrow to sites of infection and inflammation. As a result, myeloid precursors are depleted [1,6]. This process enhances myelopoiesis in the bone marrow to generate more myeloid precursors [1,7]. In this inflammatory microenvironment, newly generated myeloid precursors are reprogrammed *via* epigenetic and transcriptional regulators to become suppressor cells instead of developing into mature cells [1,8]. These myeloid-derived suppressor cells include granulocytic and monocytes that increase during mouse and human sepsis [5,9,10]. MDSCs sustain the protracted immune suppression phase of sepsis, as they produce anti-inflammatory mediators, such as TGFβ, IL-1, IL-10, and arginase, anti-inflammatory cytokines limit cell proliferation and effector functions, and expand and activate Treg cells [11,12].

We reported that calcium-binding protein S100A9 promotes MDSC development during murine sepsis [13]. Mice lacking the s100a9 gene do not generate MDSCs or develop late/protracted sepsis [13]. Under normal conditions, S100A9 is expressed in immature myeloid cells, but its levels decline with myeloid differentiation and maturation [14]. Notably, S100A9 expression increases in circulating and activated monocytes and neutrophils [15,16]. S100A9 protein is present mainly in the cytosol. It is released/secreted during infection *via* inflammation-derived signals [17,18] and functions as a proinflammatory mediator to activate immune cells and to promote neutrophil recruitment to sites of infection and inflammation, thereby amplifying the inflammatory response [15,19,20]. Importantly, we found that the S100A9 protein translocates to and accumulates in the nucleus in MDSCs in mice and humans during protracted sepsis [13].

Hotairm1 is a long non-coding RNA linked to myeloid cell development and differentiation [21,22]. We found that levels of Hotairm1 transcripts are elevated in MDSCs from mice and humans with late sepsis [23], concurrent with the accumulation of S100A9 protein in the nucleus [13]. Notably, the knockdown of Hotairm1 in late sepsis MDSCs results in the re-relocalization of S100A9 in the cytosol [23], suggesting that Hotairm1 may facilitate S100A9 nuclear localization in MDSCs during sepsis.

In the present study, we investigated how Hotairm1 affects the subcellular localization of S100A9 protein in MDSCs. Our results suggest that Hotairm1 dysregulates S100A9 protein function and activity by phosphorylation, which could inform sepsis therapeutic targeting.

## MATERIALS AND METHODS

### Mice

Male C57BL/6 mice aged 8–10 weeks were purchased from the Jackson Laboratory (Bar Harbor, ME) and housed in a pathogen-free facility. All experiments were conducted according to National Institutes of Health guidelines and approved by the East Tennessee State University Animal Care and Use Committee.

Several clinical and experimental studies indicate that cell-mediated immune responses decrease in males and increase in females during sepsis [24,25]. The female mouse is more immunologically competent than the male mouse in surviving CLP insult [26]. MDSCs suppress both innate and adaptive immune responses. Therefore, we used male mice to assess the maximal immunosuppressive effects of MDSCs on sepsis outcomes.

### Sepsis

Polymicrobial sepsis was induced by Cecal Ligation and Puncture (CLP), as previously described [27]. The cecum was twice punctured with a 23-gauge needle, and extruding feces into the abdominal cavity modeled sepsis. Mice received (i.p.) 1 ml saline for fluid resuscitation. To establish intra-abdominal infection and approximate the clinical condition of human sepsis [28], mice received subcutaneous antibiotic injections (imipenem; 25 mg/kg body weight) in 0.9% saline at 8 and 16 h after CLP. These manipulations result in early/acute sepsis and late/post-sepsis phases, with high mortality (^~^60%–70%) during the late sepsis phase [9]. The mice were followed for 28 days. Mice moribund during early sepsis (defined as the first five days after CLP) or late/protracted sepsis (days 7–28) [27] were euthanized and analyzed. Mice are moribund if they experience hypothermia (<34°C) or loss of righting reflex. A corresponding number of control/sham mice were analyzed at the same time points.

### Sepsis patients

The Institutional Review Board at the East Tennessee State University College of Medicine approved the study (IRB#:0714.6s). Patients 18 years or older admitted to Johnson City Medical Center and Franklin Woods Community Hospital in Johnson City, Tennessee, with sepsis or septic shock participated in the study. Patients were diagnosed with sepsis or septic shock using the Sepsis-3 definitions established by the 2016 International Sepsis Definitions Conference [29]. Sepsis patients were identified based on documented or suspected infection and an acute increase of ≥ 2 points in the Sequential Organ Failure Assessment (SOFA) score. The baseline SOFA score is assumed to be zero in patients without preexisting organ dysfunctions, which are determined by PaO2, platelets count, Glasgow Coma Scale score, creatinine, and bilirubin levels. Septic shock patients presented with persistent hypotension requiring vasopressors to maintain MAP ≥ 65 mm Hg and had serum lactate >2 mmol/L despite adequate fluid resuscitation [29]. Patients were presented with infections related to gram-negative or gram-positive bacteria, and the primary sites of infection included the urinary tract, circulation, and respiratory tract. Patients had at least 1 comorbid condition, such as nephropathy, psoriasis, splenectomy, colon cancer, or pulmonary disease. Patients with leukopenia due to chemotherapy, glucocorticoid therapy, or HIV infection were excluded. Patients were in two categories: early sepsis and late sepsis (i.e., protracted sepsis), based on the time of diagnosis. The early sepsis group within 1–5 days of sepsis diagnosis and late sepsis more than six days. Late sepsis blood was procured on days 6–56 after sepsis diagnosis. Blood samples from healthy control subjects were supplied by BioIVT (Gray, TN). A total of 20 sepsis patients are included in this study. Signed informed consent was obtained from all participants.

### MDSCs

Gr1^+^CD11b^+^ MDSCs were isolated from mouse bone marrow by negative selection using an EasySep mouse MDSC isolation kit according to the manufacturer’s protocol (Cat #19867; Stemcell Technologies, Cambridge, MA). The femur bone marrow was flushed out with RPMI-1640 medium (Cytiva, Marlborough, MA) under aseptic conditions. A single-cell suspension was made by filtering through a 70 μm mesh nylon strainer, followed by incubation with erythrocyte lysis buffer and washing with PBS. The cell suspension was incubated with a biotin-coupled antibody cocktail (binds to all non-Gr1^+^/CD11b^+^ cells) at room temperature for 10 min, followed by adding streptavidin-coated magnetic beads and incubating at room temperature for 5 min. Sample tubes were placed into a magnet for 3 min, and the enriched (flow-through) cell suspension containing Gr1^+^CD11b^+^ cells was transferred to a fresh tube. For cell culture, the Gr1^+^CD11b^+^ cells were incubated with RPMI-1640 medium supplemented with 100 U/ml penicillin, 100 μg/ml streptomycin, two mM L-glutamine, and 10% fetal bovine serum (R&D Systems, Minneapolis, MN) at 37°C and 5% CO_2_.

For human MDSCs, peripheral blood mononuclear cells were isolated from whole blood by density gradient centrifugation using Ficoll-Paque Plus according to the manufacturer’s protocol (G.E. Healthcare Life Sciences, Marlborough, MA) and depleted of HLA-DR^+^ cells using a biotin-coupled anti-HLA-DR antibody (Cat #13-9956-82, eBioscience, San Diego, CA) and anti-biotin microbeads (Miltenyi Biotec, Gaithersburg, MD). Next, the remaining cells were subjected to positive selection using biotin-coupled antibodies against CD33 (Cat #MA1-19522; Invitrogen, Waltham, MA), CD11b (Cat #130-113-795), and LOX-1 (Cat #130-122-119; both from Milteny Biotec).

To inhibit KDM6A demethylase activity, GSK-J4 HCl (Cat #S7070, Selleckchem, Houston, TX) was reconstituted in DMSO and used at 4 μM final concentration.

### RNA immunoprecipitation

Protein-RNA co-immunoprecipitation detected Hotairm1 RNA binding to S100A9 protein according to a previously published method [30], with some modifications. Gr1^+^CD11b^+^ cells (~10 × 10^6^) were washed with warm PBS and incubated with 0.2% formaldehyde (in PBS) for 10 min at room temperature to preserve RNA-protein complexes. The cross-linked cells were incubated for 30 min on ice in cell lysis buffer (Cat #87788; Thermo Fisher Scientific, Waltham, MA) containing 30 U/ml RNase inhibitor and 1x protease inhibitor cocktail. The cell lysate was cleared by centrifugation at 10,000 rpm for 15 min at 4°C. The supernatant was incubated with 5 μg of antibody specific to S100A9 (Cat #sc-58706; Santa Cruz Biotechnology, Dallas, TX), phospho-S100A9/threonine113 (Cat #PA5-64853; Invitrogen), or IgG isotype control (Cat #10400C; Invitrogen) and 25 μl of protein G-coated magnetic beads (Bio-Rad) overnight at 4°C with rotation. The samples were treated with DNase I for 10 min at 37°C. The beads, with the attached RNA-protein complexes, were recovered and washed with TBS-T buffer. RNA was extracted from the immunoprecipitated complex by Trizol reagent (Invitrogen) and used to detect Hotairm1 by RT-qPCR. In some experiments, the immunoprecipitated complexes were assessed by immunoblotting using anti-phospho-S100A9 or anti-S100A9 antibody.

### Cell transfection

For Hotairm1 knockdown, Gr1^+^CD11b^+^ cells were transfected with a pool of Hotairm1-specific or control siRNA (Qiagen, Germantown, MD). The siRNA mixture was suspended in HiPerFect reagent (Qiagen) at a 0.5 μM final concentration. The cells were transfected and incubated with RPMI-1640 medium for 36 h.

For Hotaim1 transfection, Hotairm1 cDNA was cloned in the pReceiver-M02 expression vector downstream of the CMV promoter. An empty control vector (pReceiver-M02CT) was a negative control (GeneCopoeia, Rockville, MD). The plasmid DNA was suspended in HiPerFect reagent at a 0.5 μg/ml final concentration (Qiagen). Gr1^+^CD11b^+^ cells were transfected and incubated with RPMI-1640 medium for 36 h.

### Western blot

Whole-cell lysates were prepared using 1x RIPA lysis buffer (Cat #20-188; Millipore-Sigma, Rockville, MD) containing 50 mM Tris-HCl [pH 7.4], 150 mM NaCl, 1% NP-40, 0.25% sodium deoxycholic acid, 1 mM EDTA (Millipore, Temecula, CA), and protease inhibitors (Thermo Fisher Scientific, Waltham, MA). Cell lysates or immunoprecipitated protein complexes were resolved onto SDS-10% polyacrylamide gel (Bio-Rad, Hercules, CA) and transferred to nitrocellulose membranes (Thermo Fisher Scientific). The membranes were blocked with 5% milk in Tris-buffered saline/Tween-20 for one h at room temperature and then probed overnight at 4°C with an antibody specific to phospho-S100A9 (Cat #PA5-64853; Invitrogen), p38 (Cat #sc-81621; Santa Cruz Biotechnology), or phospho-p38 (Cat #690202; BioLegend). After washing, the blots were incubated with the appropriate HRP-conjugated secondary antibody for 2 h at room temperature. Proteins were detected with the enhanced chemiluminescence detection system (Thermo Fisher Scientific). The protein bands were visualized using the ChemiDoc XRS System (Bio-Rad), and the images were captured with the Image Lab Software V3.0. The membranes were stripped and reprobed for β-actin (Invitrogen).

### Real-time PCR

Quantitative Real-Time PCR (RT-qPCR) was performed to measure Hotairm1 transcripts in S100A9-immunoprecipitated protein complexes. RNA was isolated from the protein complexes using TRIzol reagent (Invitrogen) and subjected to reverse transcription using QuantiTect Reverse Transcription kit (Qiagen). The cDNA was amplified by qPCR using QuantiTect SYBR Green PCR Master Mix kit and pre-designed lncRNA qPCR Primer Assays specific to mouse Hotairm1 (assay I.D. #LPM17359A) and human Hotairm1 (assay I.D. #LPH10483A) (Qiagen). Values were normalized to the IgG immunoprecipitated samples.

### ELISA

Enzyme-linked immunosorbent assay was performed to determine the levels of mouse and human IL-10 in the culture supernatants using specific ELISA kits (BioLegend) according to the manufacturer’s instructions.

### Statistical analysis

Data were analyzed with Microsoft Excel. Values are expressed as mean ± S.D. Differences between two groups were determined by a two-tailed student’s t-test. P-values of <0.05 were considered statistically significant.

## RESULTS

### Hotairm1 binds S100A9 protein in MDSCs during late sepsis in mice

During late sepsis, S100A9 protein moves from the cytosol to the nucleus in Gr1^+^CD11b^+^ MDSCs, concurrent with increases in Hotairm1 transcripts, and knockdown of Hotairm1 results in the shuttling of S100A9 protein back to the cytosol [23]. Since most immune-related lncRNAs regulate cellular processes through interaction with their target proteins, we asked whether Hotairm1 controls S100A9 protein localization in MDSCs *via* post-translational protein modification. We previously reported that in early sepsis Gr1^+^CD11b^+^ cells, S100A9 protein is mainly detected in the cytosol in a phosphorylated form (phosphorylated on threonine number 113). In contrast, in late sepsis Gr1^+^CD11b^+^ cells, S100A9 is present mainly in the nucleus in an unphosphorylated form [23]. To determine the role of Hotairm1 in S100A9 protein phosphorylation, we performed protein-RNA co-immunoprecipitation using protein extracts from Gr1^+^CD11b^+^ cells with anti-S100A9 and anti-phospho-S100A9 antibodies.

**Figure 1:**
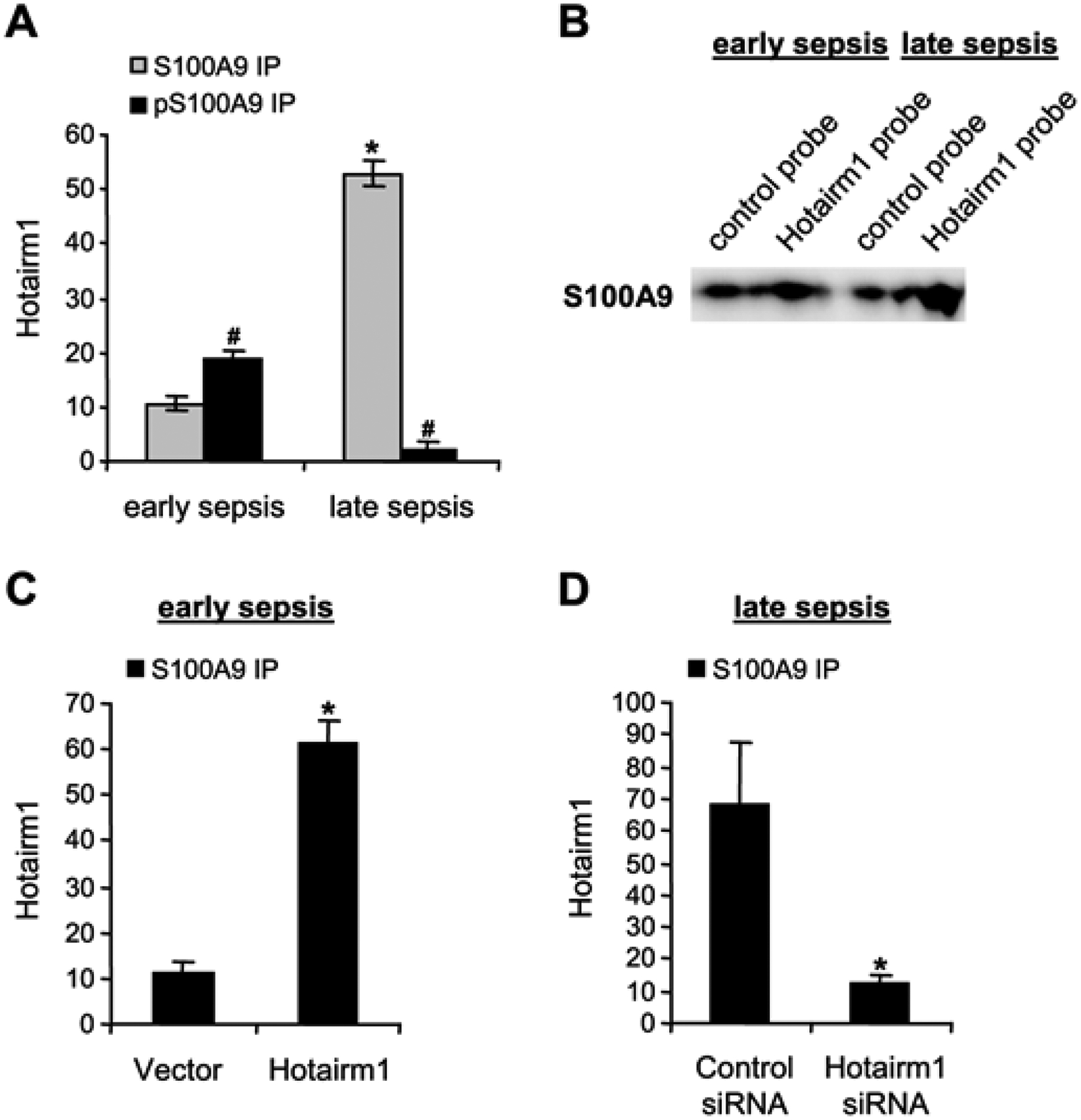
Binding of Hotairm1 to S100A9 protein in Gr1^+^CD11b^+^ cells during sepsis. (A) Hotairm1 binding to S100A9 protein increases during late sepsis. Gr1^+^CD11b^+^ cells were purified from the bone marrow of septic mice using magnetic beads and anti-Gr1 and anti-CD11b antibodies. The cells were washed and fixed in 0.2% formaldehyde to preserve protein-RNA complexes. The whole-cell lysate was prepared and cleared by centrifugation. The supernatant was incubated with 5 μg of S100A9, phospho-S100A9 (Thr113), or IgG isotype control antibody and then cross-linked to protein G magnetic beads overnight at 4°C. The beads were washed and RNA was extracted from the immunoprecipitated protein-RNA complexes by Trizol reagent and used to detect Hotairm1 by RT-qPCR, using the Hotairm1 primer assay. (B) Hotairm1-S100A9 binding specificity was determined ex vivo using an RNA-protein pull-down assay kit (Pierce). Gr1^+^CD11b^+^ cells were isolated from septic mice. A biotin-labeled Hotarm1 probe was incubated with streptavidin magnetic beads in RNA-capture buffer for 30 min at room temperature. The RNA-bound beads were washed and incubated with Gr1^+^CD11b^+^ cell lysate in RNA-protein binding buffer at 4°C for 1 h. The bead-bound RNA with the associated protein complexes was run onto a polyacrylamide gel. The gel was blotted, and the membrane was probed with an S100A9 antibody. The results are representative of two experiments. (C) Gr1^+^CD11b^+^ cells were isolated from early septic mice and transfected with control or Hotairm1 vector for 36 h. The cells were treated, and protein-RNA complexes were immunoprecipitated with anti-S100A9 or IgG antibody as described in *A*. RNA was extracted from the immunoprecipitated protein-RNA complexes by Trizol reagent, and levels of Hotairm1 bound to S100A9 protein were determined by RT-PCR. (D) Gr1^+^CD11b^+^ cells were isolated from late septic mice and transfected with control/scramble or Hotairm1 siRNA for 36 h. The cells were treated, and protein-RNA complexes were immunoprecipitated with anti-S100A9 or anti-IgG antibodies as described in *A*. RT-PCR determined the levels of Hotairm1 bound to S100A9 protein. Values were normalized to the IgG immunoprecipitated samples. The data are mean ± SD of 5 mice per group.*p < 0.05, *vs*. early sepsis (*A*), vector (*C*), control siRNA (*D*); #p < 0.05, *vs*. S100A9 IP. IP., immunoprecipitation.

Real-time PCR analysis of the immunoprecipitated RNA showed Hotairm1 binding to S100A9 and phospho-S100A9 proteins at varying levels in Gr1^+^CD11b^+^ cells from mice with early sepsis (Figure 1A). Notably, Hotairm1 transcripts were detected in the S100A9 protein complex at a significantly higher level in late sepsis Gr1^+^CD11b^+^ cells. Next, we used a biotin-labeled Hotairm1 synthetic probe to confirm Hotairm1 binding to the S100A9 protein. The RNA probe was incubated with Gr1^+^CD11b^+^ cell extract and then pulled down with streptavidin beads. Western blot analysis of the bead-bound RNA-protein complexes showed increased S100A9 protein binding to Hotairm1 in late sepsis Gr1^+^CD11b^+^ cells (Figure 1B). These results show that Hotairm1 RNA binds mainly to the unphosphorylated S100A9 protein in MDSCs during late sepsis.

### Hotairm1 inhibits S100A9 protein phosphorylation in MDSC during late sepsis

Hotairm1 transcripts are detected at low levels in early sepsis Gr1^+^CD11b^+^ cells and are significantly increased in late sepsis [23]. We determined whether manipulating Hotairm1 levels in Gr1^+^CD11b^+^ cells affects the Hotairm1-S100A9 binding. To this end, Gr1^+^CD11b^+^ cells from early septic mice were transfected with Hotairm1 plasmid and cultured for 36 h. In parallel, Gr1^+^CD11b^+^ cells from late septic mice, where the level of Hotairm1 is highest, were transfected with Hotairm1 siRNA to knock down Hotairm1. The Hotairm1-S100A9 protein complexes were pulled down from the whole cell lysate using the S100A9 antibody. PCR analysis of the immunoprecipitated RNA showed that increasing Hotairm1 transcripts in early sepsis Gr1^+^CD11b^+^ cells increased the amount of S100 protein binding, whereas Hotairm1 knockdown in late sepsis Gr1^+^CD11b^+^ cells significantly reduced S100A9 binding (Figures 1C and 1D).

### Hotairm1 prevents p38 MAPK binding to S100A9 protein in MDSCs during late sepsis

**Figure 2:**
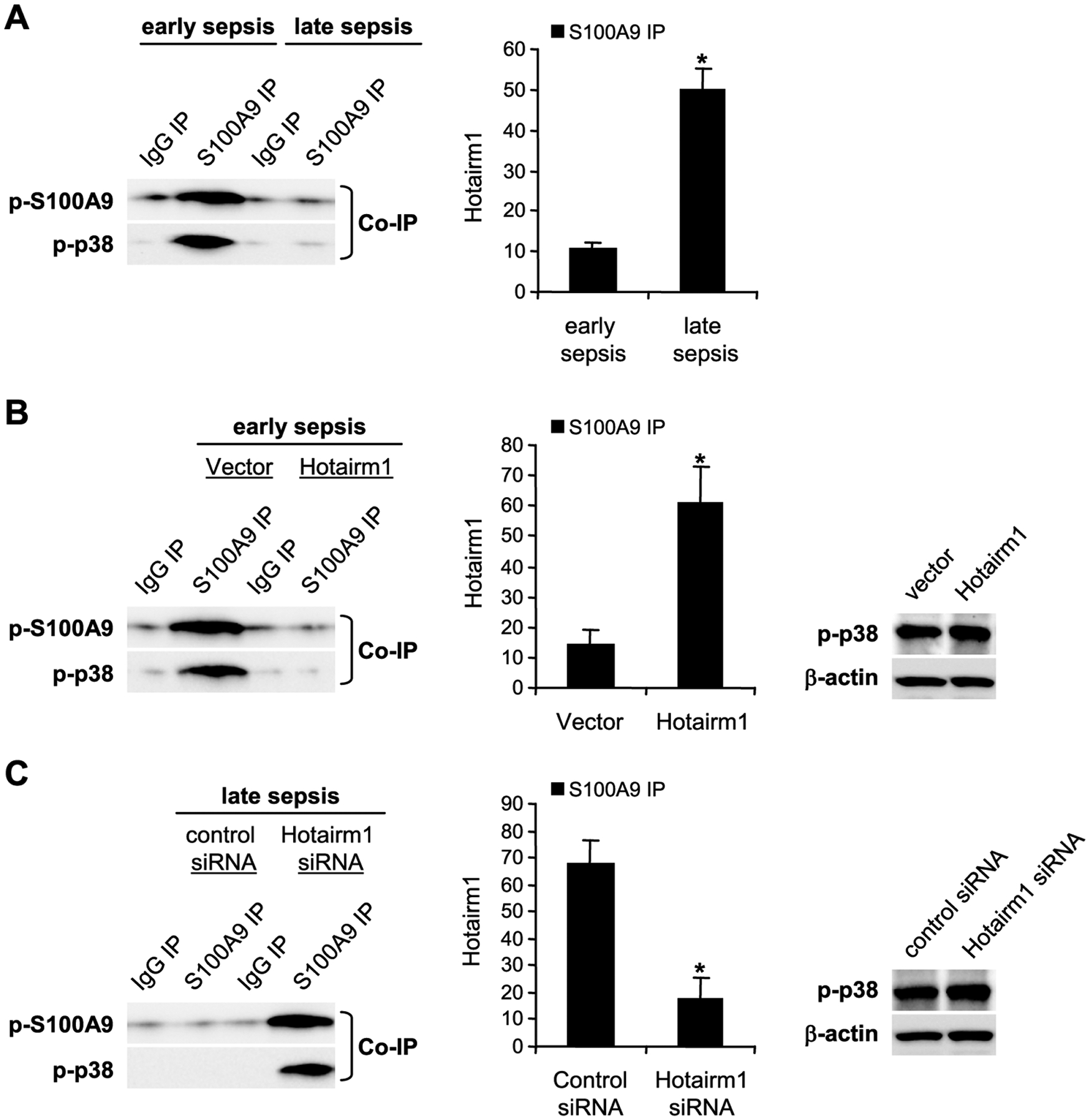
Hotairm1 prevents S100A9 protein phosphorylation during late sepsis. (A) Gr1^+^CD11b^+^ cells were purified from bone marrow using magnetic beads and anti-Gr1 and anti-CD11b antibodies. The cells were fixed in 0.2% formaldehyde, and the whole cell lysate was prepared and cleared by centrifugation. The supernatant was incubated with anti-S100A9 or anti-IgG isotype control antibody and then cross-linked to protein G magnetic beads overnight at 4°C. The co-immunoprecipitated protein-RNA complexes were assessed by immunoblotting using phospho-S100A9 antibody. The membrane was stripped and reprobed with a phospho-p38 antibody. In other experiments, RNA was extracted from the bead-bound protein-RNA complexes by Trizol reagent and used for detecting Hotairm1 by RT-qPCR, using the Hotairm1 primer assay. (B) Gr1^+^CD11b^+^ cells were transfected with control vector or Hotairm1 plasmid for 36 h. (C) Gr1^+^CD11b^+^ cells were transfected with control/scramble or Hotairm1 siRNA for 36 h. A portion of the cells was used to assess phospho-p38 protein levels by Western blot (right panels). The remainder of the cells was treated, and protein-RNA complexes were immunoprecipitated with S100A9 or IgG control antibody as described in *A*. The co-immunoprecipitated protein complexes were immunoblotting using phospho-S100A9 antibody. The membranes were stripped and reprobed with phospho-p38 antibody. Western blot results are representative of two experiments. The data are mean ± SD of 5 mice per group.*p < 0.05.

In myeloid cells, S100A9 is phosphorylated in the cytosol on threonine number 113 by p38 MAPK, which promotes its translocation to the plasma membrane and subsequent release from the cell [31]. Notably, phosphorylation of the S100A9 protein is diminished in Gr1^+^CD11b^+^ cells during the late sepsis phase [13]. We performed protein-RNA co-immunoprecipitation and PCR analysis to determine how Hotairm1 inhibits S100A9 phosphorylation in Gr1^+^CD11b^+^ cells. Whole-cell lysates were immunoprecipitated with S100A9 antibody, and the immunoprecipitated protein complexes were immunoblotted with phospho-S100A9 antibody and reprobed with phospho-p38 antibody. Both phospho-S100A9 and phospho-p38 proteins were present in the same protein complex in early sepsis Gr1^+^CD11b^+^ cells and almost diminished during late sepsis (Figure 2A, left panel). The inhibition of S100A9 phosphorylation in late sepsis Gr1^+^CD11b^+^ cells mirrored the significant increases in Hotairm1 binding to S100A9 protein, as demonstrated by the increase in Hotairm1 transcripts in the immunoprecipitated protein complex (Figure 2A).

To determine whether the increase in Hotairm1 RNA in late sepsis Gr1^+^CD11b^+^ cells is responsible for the decrease in S100A9 phosphorylation by p38 MAPK, we increased Hotairm1 transcripts in early sepsis Gr1^+^CD11b^+^ cells *via* transfection with Hotairm1 plasmid and then examined phospho-S100A9 and phospho-p38 protein levels. Protein-RNA co-immunoprecipitation and real-time PCR analysis of the immunoprecipitated RNA showed that increasing levels of Hotairm1 RNA disrupted S100A9 phosphorylation by p38 MAPK (Figure 2B, left panel). The decrease in S100A9 phosphorylation was not due to a decrease in phospho-p38 because Western blotting showed phosph-p38 level was not changed after Hotairm1 plasmid transfection (Figure 2B).

Additionally, we performed protein-RNA co-immunoprecipitation and PCR analysis in late sepsis Gr1^+^CD11b^+^ cells following Hotairm1 knockdown by siRNA. Figure 2C shows that decreasing Hotairm1 transcripts resulted in remarkable increases in S100A9 phosphorylation, as the amounts of phospho-S100A9 and phospho-p38 proteins increased in the co-immunoprecipitated protein complex.

These results support that S100A9 protein is phosphorylated by p38 MAPK in Gr1^+^CD11b^+^ cells and that the increase in Hotairm1 levels during late sepsis prevents S100A9 phosphorylation.

### Knockdown of Hotairm1 transcripts in late sepsis Gr1^+^CD11b^+^ cells attenuates IL-10 production

**Figure 3:**
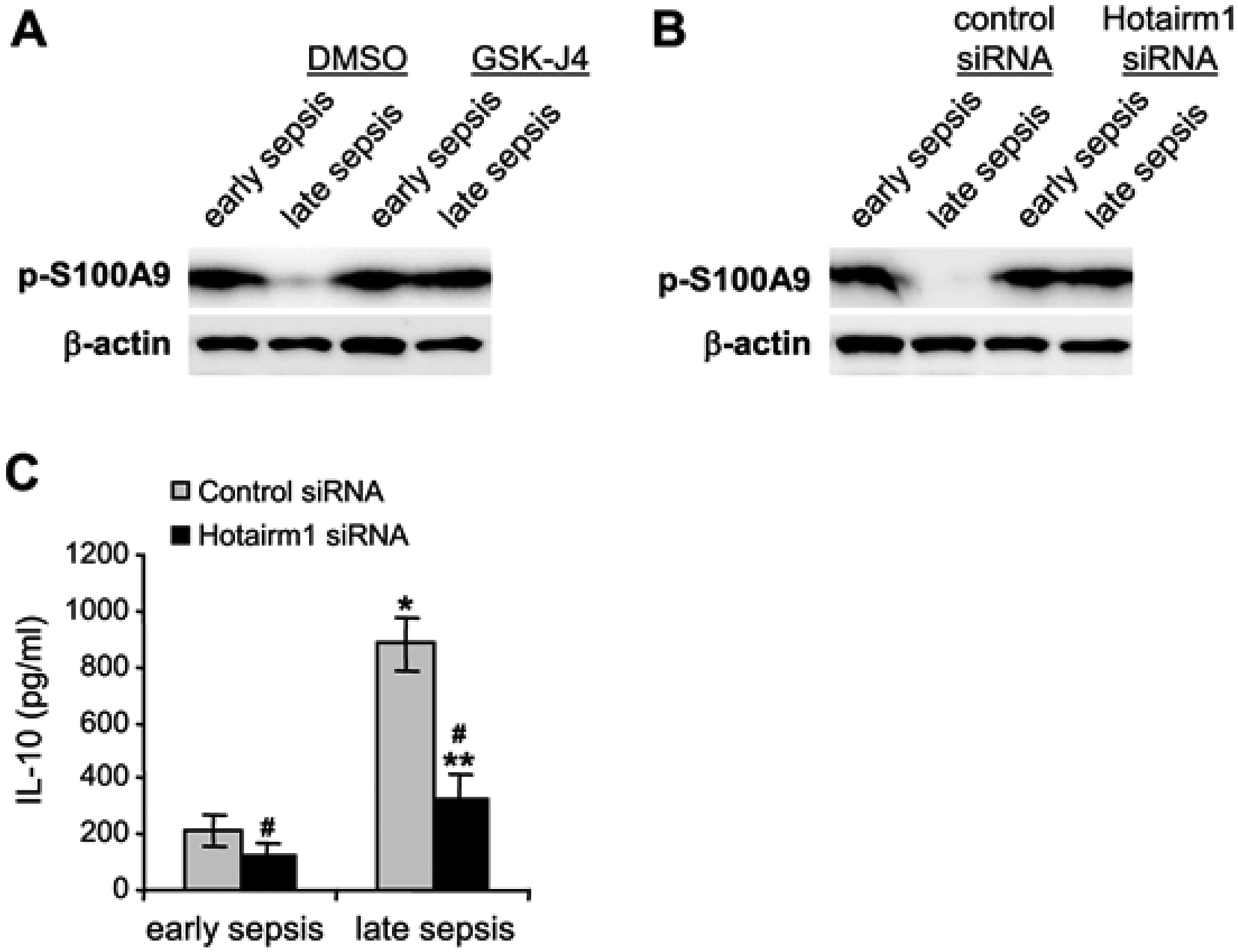
Depleting Hotairm1 restores S100A9 phosphorylation during late sepsis. Gr1^+^CD11b^+^ cells were purified from bone marrow using magnetic beads and anti-Gr1 and anti-CD11b antibodies. (A) The cells were incubated with 4 μM of GSK-J4 or 0.1% DMSO as a control for 12 h. Whole-cell lysates was resolved onto a polyacrylamide gel and immunoblotted with phospho-S100A9 antibody. (B) Gr1^+^CD11b^+^ cells were transfected with control/scramble or Hotairm1 siRNA for 36 h. The levels of phospho-S100A9 protein were determined as in *B*. The results are representative of two experiments. (C) The cells were transfected as in B. After 36 h, the cells were washed and incubated with 1 μg/ml of bacterial lipopolysaccharide (LPS, E. coli, serotype 0111:B4; Sigma, St. Louis, MO) for 12 h to stimulate IL-10 production. ELISA determined the levels of IL-10 in the culture supernatants. Samples were run in duplicate. The data are mean ± SD of 5 mice per group.*p < 0.05, *vs*. control early sepsis or Hotairm1 siRNA; **p < 0.05, *vs*. Hotairm1 siRNA/early sepsis; #p < 0.05, *vs*. control siRNA.

Hotairm1 transcription in Gr1^+^CD11b^+^ cells during sepsis is regulated by an epigenetic mechanism that involves histone methylation and binding of transcription factor PU.1 at its proximal promoter [32]. In early sepsis, the Hotairm1 promoter is enriched with the transcriptionally repressive histone mark H3K27me3, which inhibits its promoter transcription by PU.1. During late sepsis, the histone demethylase KDM6A catalyzes the removal of H3K27me3, leading to PU.1 binding and activation of Hotairm1 transcription. We reported that chemical inhibition of KDM6A by GSK-J4 significantly reduces Hotairm1 transcripts in late sepsis Gr1^+^CD11b^+^ cells [32]. Because siRNA-mediated knockdown of Hotairm1 in late sepsis Gr1^+^CD11b^+^ cells increased S100A9 phosphorylation by p38 MAPK (Figure 2C), we investigated whether inhibition of KDM6A demethylase activity can affect S100A9 protein phosphorylation. We treated Gr1^+^CD11b^+^ cells with 4 μM of GSK-J4 for 12 h. Western blotting using whole cell extracts showed that treatment with GSK-J4 remarkably increased levels of phospho-S100A9 protein in late sepsis Gr1^+^CD11b^+^ cells (Figure 3A). Notably, this increase in phospho-S100A9 levels was similar to the increase in S100A9 phosphorylation following Hotairm1 knockdown (Figure 3B).

Late sepsis Gr1^+^CD11b^+^ cells produce increased amounts of the immunosuppressive IL-10 cytokine [9]. We determined whether the increase in S100A9 phosphorylation following Hotairm1 knockdown decreased IL-10 levels in late sepsis Gr1^+^CD11b^+^ cells. As shown in Figure 3C, Hotairm1 knockdown significantly decreased the LPS-induced production of IL-10. These results support that increasing S100A9 phosphorylation in late sepsis Gr1^+^CD11b^+^ cells *via* targeting Hotairm1 attenuates their immunosuppressive function.

### Hotairm1 controls S100A9 protein phosphorylation in MDSCs during human sepsis

**Figure 4:**
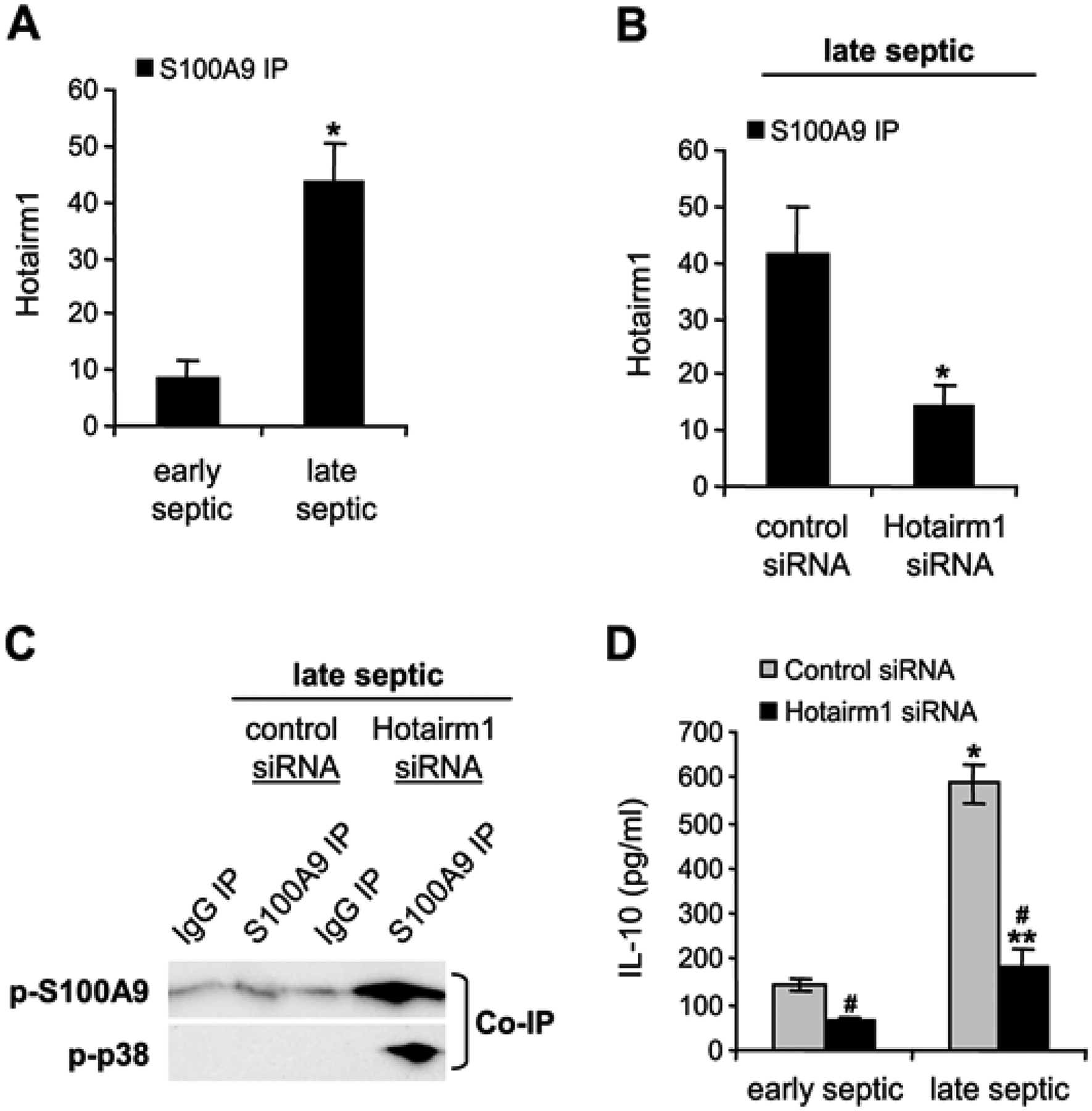
Hotairm1 binding to S100A9 protein in MDSCs from septic patients. PBMCs were isolated from early and late septic patients and depleted of the HLA-DR^+^ cells using biotin-coupled anti-HLA-DR antibody and anti-biotin microbeads, followed by the positive selection of CD33^+^LOX1^+^ cells with biotin-coupled anti-CD33, anti-CD11b, and anti-LOX1 antibodies. (A) The CD33^+^CD11b^+^LOX1^+^HLA-DR- cells were fixed in 0.2% formaldehyde to preserve RNA-protein complexes. The whole cell lysate was incubated with 5 μg of anti-S100A9 or anti-IgG isotype control antibody and then cross-linked to protein G magnetic beads overnight at 4°C. RNA was extracted from the immunoprecipitated protein-RNA complexes by Trizol reagent and used for detecting Hotairm1 by RT-qPCR, using human Hotairm1 primer assay. (B) CD33^+^CD11b^+^LOX1^+^HLA-DR- cells from late septic patients were transfected with scramble or Hotairm1 siRNA for 36 h. The cells were treated as described in *A*, and RT-PCR determined levels of Hotairm1. (C) Whole cell lysate of CD33^+^CD11b^+^LOX1^+^HLA-DR-cells were cleared by centrifugation and subjected to immunoprecipitation with S100A9 or IgG isotype control antibody as described in *A*. The immunoprecipitated protein complexes were assessed by immunoblotting using phospho-S100A9 (Thr113) antibody. The membrane was stripped and reprobed with a phospho-p38 antibody. The results are representative of two experiments. (D) CD33^+^CD11b^+^LOX1^+^HLA-DR-cells were transfected with scramble or Hotairm1 siRNA for 36 h, then washed and incubated with 1 μg/ml of bacterial LPS for 12 h. ELISA determined the levels of IL-10 in the culture supernatants. The data are mean ± SD of 5 patients per group.*p < 0.05, *vs*. early septic or control siRNA; **p < 0.05, *vs*. Hotairm1 siRNA/early septic; #p < 0.05, *vs*. control siRNA.

Levels of Hotairm1 transcripts significantly increase in MDSCs in septic patients who develop late/protracted sepsis state, concurrent with an accumulation of unphosphorylated S100A9 protein in the nucleus [23]. Protein-RNA co-immunoprecipitation and PCR, analysis of the immunoprecipitated RNA, showed low levels of Hotairm1 RNA binding to S100A9 protein in MDSCs from early septic patients, and the amount of Hotairm1 binding significantly increased during late sepsis (Figure 4A). In addition, the amount of Hotairm1 transcripts associated with the unphosphorylated S100A9 protein significantly decreased following Hotairm1 knockdown in late sepsis MDSCs (Figure 4B).

We next assessed the levels of S100A9 protein phosphorylation following Hotairm1 knockdown in MDSCs isolated from late septic patients. Cell extract was immunoprecipitated with S100A9 antibody, and the immunoprecipitated protein complexes were probed with phospho-S100A9 protein and then reprobed with phospho-p38 protein. As shown in Figure 4C, phospho-S100A9, and phospho-p38 proteins were barely detected in the knockdown control cells. Following Hotairm1 knockdown, the amount of phospho-S100A9 protein remarkably increased. In addition, we investigated the effect of Hotairm1 knockdown on IL-10 production following stimulation with bacterial LPS. Hotairm1 knockdown significantly reduced IL-10 levels in MDSCs (Figure 4D). These results support that Hotairm1 binds S100A9 protein in MDSCs during human sepsis and prevents phosphorylation by p38 MAPK during late sepsis. These results also suggest that targeting Hotairm1 can attenuate MDSC suppressive function.

## DISCUSSION

**Figure 5:**
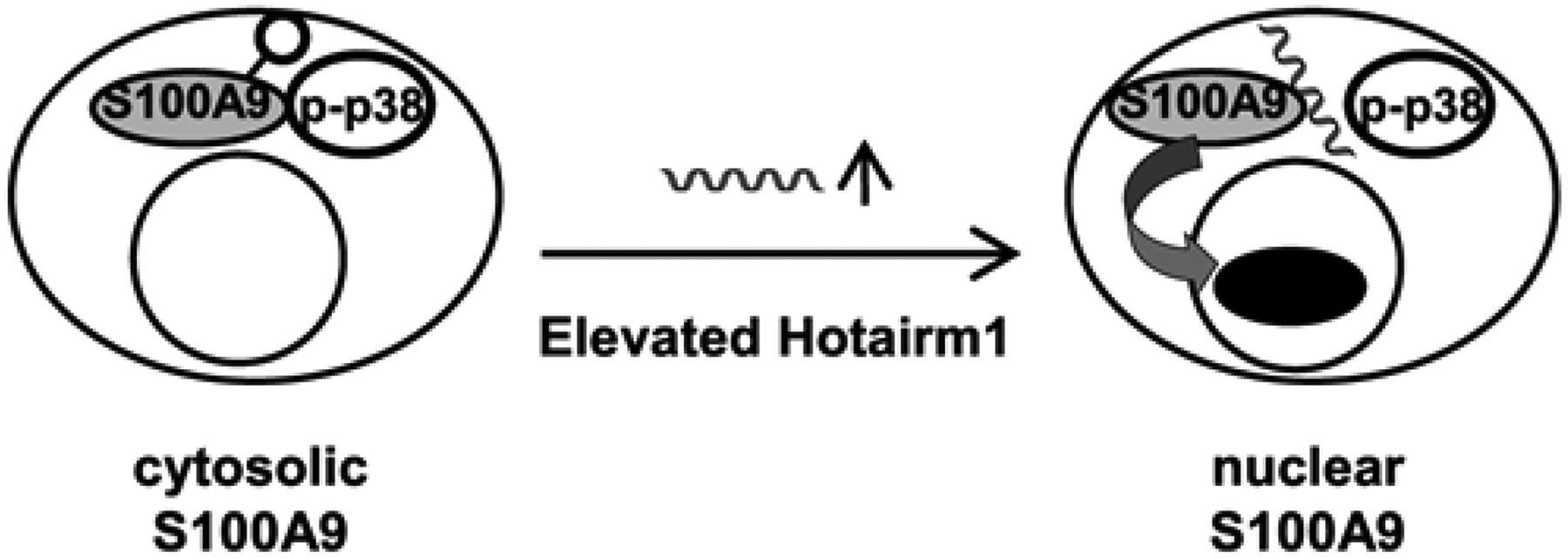
A proposed model for regulation of S100A9 protein location by Hotairm1 in MDSCs during sepsis. In early sepsis, S100A9 is phosphorylated on threonine 113 by p38 MAPK and resides in the cytosol. As sepsis progresses to the late/chronic state, Hotairm1 is increased and binds to S100A9 protein and prevents its phosphorylation, resulting in translocation to the nucleus to support MDSC immunosuppressive phenotype.

The present study shows that Hotairm1 prevents the phosphorylation of S100A9 protein in MDSCs in mice and humans with protracted sepsis. S100A9 protein expression increases in Gr1^+^CD11b^+^ myeloid cells during sepsis but accumulates in the nucleus in an unphosphorylated form as sepsis shifts to the late/protracted state [13]. S100A9 is phosphorylated by p38 MAPK in the cytosol, which facilitates its translocation to the plasma membrane for secretion, to act as a soluble proinflammatory mediator [15,18,31]. Accumulation of S100A9 protein in the unphosphorylated form in the nucleus in Gr1^+^CD11b^+^ cells during late sepsis reprograms them to the immunosuppressive Gr1^+^CD11b^+^ phenotype (i.e., MDSC) [13]. We found that depletion of Hotairm1 from late sepsis MDSCs from mice and humans restores levels of phospho-S100A9 protein. These findings support that Hotairm1 modifies S100A9 protein localization and function *via* post-translational protein modifications during late sepsis (Figure 5).

Our results suggest that Hotairm1 disrupts the p38 MAPK-mediated phosphorylation of S100A9 in MDSCs. Hotairm1 was co-immunoprecipitated with unphosphorylated S100A9 protein, suggesting that Hotairm1 binding displaces p38 MAPK from the S100A9 protein complex. Hotairm1 RNA is detected in Gr1^+^CD11b^+^ cells at low levels during early sepsis, but it increases significantly during late sepsis [23], concurrently with the accumulation of unphosphorylated S100A9 protein in the nuclear compartment [13]. Increasing Hotairm1 transcripts in early sepsis Gr1^+^CD11b^+^ cells diminished S100A9 phosphorylation by p38 MAPK. Conversely, the knockdown of Hotairm1 in late sepsis Gr1^+^CD11b^+^ MDSCs increased S100A9 protein phosphorylation. Since the S100A9 protein is released from the cytosol upon phosphorylation and secreted as a soluble mediator to promote inflammation [33], our study suggests that Hotairm1 modifies the S100A9 function by primarily targeting phosphorylation.

Recent studies have shown that sepsis induces epigenetic processes in bone marrow cells that can cause changes in cell phenotypes. For example, epigenetic modifications in bone marrow progenitors can lead to the development of impaired monocytes/macrophages [33,34].

We have previously reported that the increase in Hotairm1 transcripts in MDSCs during late sepsis emerges from epigenetic modifications at its proximal promoter [35]. In early sepsis Gr1^+^CD11b^+^ cells, the Hotairm1 promoter is enriched with the repressive transcription histone mark H3K27me3, which inhibits its promoter transcription. During late sepsis, histone lysine demethylase KDM6A removes H3K27me3, increasing the transcription activation marks H3K4me3 and subsequently activating its transcription by PU.1 [35]. Inhibiting KDM6A demethylase activity with its specific inhibitor GSK-J4 in late sepsis Gr1^+^CD11b^+^ cells significantly reduces Hotairm1 levels [32]. The current study showed that treatment of Gr1^+^CD11b^+^ cells from late septic mice with GSK-J4 increased the levels of phospho-S100A9 protein. Importantly, this increase in S100A9 phosphorylation mirrored the increase in phospho-S100A9 levels following the Hotairm1 knockdown. These findings support that the Hotairm1-mediated inhibition of S100A9 phosphorylation in MDSCs during late sepsis is part of an epigenetic mechanism that promotes MDSC development.

Many patients who survive acute sepsis develop a Chronic Critical Illness (CCI) phenotype characterized by persistent low-grade inflammation and chronic immunosuppression, also called “persistent inflammatory, immunosuppressive, and Protein Catabolic Syndrome (PCS)” [1,5]. MDSCs contribute to the immunopathology of this CCI phenotype, which is observed in about 30% of sepsis survivors [1,10]. Expansion of MDSCs is associated with an increased risk of secondary infections and elevated mortality in patients with chronic CCI [10,36]. MDSCs suppress the immune response through various mechanisms, including inhibition of effector T cells, inhibition of macrophage and dendritic cell functions, stimulation of immunosuppressive Treg cell expansion, and production of immunosuppressive cytokines such as IL-10 [11,37]. IL-10 promotes sepsis-induced immunosuppression [38] and supports S100A9 protein translocation to the nucleus in mouse MDSCs during late sepsis [39].

Our findings that Hotairm1 knockdown in late sepsis MDSCs from mice and humans increased S100A9 phosphorylation and reduced IL-10 production are biologically significant. We have previously shown that IL-10 production is associated with MDSC accumulation in mice during late sepsis [9,33] and that mice lacking the S100a9 gene do not generate immunosuppressive MDSCs and have significantly less IL-10 [13]. Although S100A9 is mainly known as a soluble inflammatory mediator/alarmin that enhances the inflammatory responses [15,19], our studies suggest that, in the context of late sepsis, Hotairm1 can switch S100A9 protein into a nuclear co-factor that supports the MDSC phenotype. Hotairm1 modifies the S100A9 protein function by preventing its phosphorylation and subsequent release. Other lncRNAs support molecular processes by modifying their target proteins. For example, lncRNA-CD promotes the differentiation of human monocytes into dendritic cells *via* binding to transcription factor p-Stat3 and preventing its dephosphorylation/inactivation by tyrosine phosphatase SHP1 [40–42].

## CONCLUSION

In summary, Hotairm1 prevents the phosphorylation of S100A9 protein in MDSCs during late sepsis. Hotairm1 knockdown increases S100A9 phosphorylation and reduces IL-10 production in mouse and human MDSCs. Phosphorylation of S100A9 supports its translocation to the plasma membrane for secretion. Since cytosol-to-nucleus translocation of S100A9 in MDSCs only occurs when S100A9 is unphosphorylated and because mice lacking S100A9 expression do not generate MDSCs, the present results support that by preventing its phosphorylation, Hotairm1 modifies S100A9 protein, turning it from a proinflammatory mediator into a chronic immune repressor. Potential impact of this study is that epigenetic targeting of Hotairm1 might alleviate the sepsis-induced immunosuppression during MDSC expansion.
